# Screening of Durum Wheat Cultivars for Selenium Response under Contrasting Environments, Based on Grain Yield and Quality Attributes

**DOI:** 10.3390/plants11111437

**Published:** 2022-05-28

**Authors:** Sourour Ayed, Imen Bouhaouel, Afef Othmani

**Affiliations:** 1 Field Crops Laboratory, LR20-INRAT-02, National Agricultural Research Institute of Tunisia, University of Carthage, Ariana 2049, Tunisia; othmaniafef@yahoo.com; 2 Genetics and Cereal Breeding Laboratory, LR14AGR01, National Agronomic Institute of Tunisia, University of Carthage, Tunis 1082, Tunisia; imenbouhaouel@gmail.com

**Keywords:** *Triticum durum*, field conditions, foliar supply, grain yield, quality attributes, selenium

## Abstract

In the literature, little information is available on the effect of Selenium (Se) on durum wheat yield and grain quality performances. A field investigation was conducted to explore the effect of exogenous Se foliar supply on two types of durum wheat germplasm; i.e., 16 advanced lines and nine modern varieties. The Se effect was assessed on grain yield as well as on technological quality traits (moisture, protein and gluten contents, Zeleny sedimentation index, and deformation energy) in two contrasting environments in Tunisia, namely Kef–Boulifa (semi-arid region) and Beja (sub-humid region). The results displayed significant effects of environments, Se foliar application, and cultivars on grain yield and quality attributes. For grain yield performance, the beneficial effect of Se was more pronounced under the Kef–Boulifa environment, and conversely for the grain quality. A genetic variation was observed within and among the two environments under both Se treatments (with and without Se). Notably, the Se-treated advanced lines displayed the highest grain yield under Kef–Boulifa and Beja conditions. Although these cultivars showed better grain quality in both sites, the modern varieties valorized the Se foliar application better. Cultivars that recorded the highest values for the studies attributes were not necessarily those that valorized the Se supply better. Interestingly, some advanced lines have noted superiority compared to the modern varieties. In this study, cultivars that combine both good yield and good grain quality were determined for semi-arid (L11, L1, Dhahbi, and Maali) and sub-humid (L2, L14, L6, L3, Salim, and INRAT 100) zones. The screening results provide genetic material that could be exploited in breeding programs to improve Se use efficiency.

## 1. Introduction

For several decades, the production of durum wheat (*Triticum turgidum* L. ssp. *durum* [Desf.] Husn.) has been mainly focused on maximizing yield under water scarcity and poor soil fertility conditions within the context of climate change scenarios, especially in developing countries of North Africa, such as Tunisia. Durum wheat is the most staple cultivated crop in this country, representing 54% of the cereal growth area (~1.5 million ha) [1,2]. Maintaining the stability of durum wheat production in Tunisia is a real challenge [3] that requires the identification of new approaches to boost durum wheat growth and production by mitigating the impact of environmental stresses [4]. In fact, developing new varieties has been achieved using several methods, including breeding for agronomic traits, the use of genetic markers, and genomic selection, with various levels of success. Recently, these breeding methods might be strengthened by eco-friendly and sustainable technologies to enhance crop performance, including the supply of mineral compounds. One of the current and interesting strategies that have recently gained attention is the use of Selenium (Se), with its various forms (selenite, selenate, organic compounds, and nano-selenium), to improve agronomic performances (growth and yield) and nutrient uptake in some plant species [5,6,7,8,9,10,11], mainly wheat [12,13] under abiotic and biotic stress conditions [14,15].

The increase in grain yield might be attributed to several Se indirect actions, such as accumulation of starch in the chloroplast [16], decrease in chlorophyll breakdown, and promotion of chlorophyll fluorescence and photosystem efficiency II (PSII) [17]. Furthermore, it can contribute to the regulation of plant water status via boosting the absorption efficiency of water and reducing the water loss through plant tissue [18]. Exo-applied Se can also regulate the activities of both enzymatic and non-enzymatic antioxidants and metabolites leading to a better capacity to scavenge reactive oxygen species (ROS) and protection of the cell membrane against lipid peroxidation, which impede plant performance, especially under stress conditions [19]. In particular, this element acts as a cofactor for antioxidant enzymes, such as glutathione peroxidase [20]. Se can also contribute to the uptake of more nutrients by plants, as reported in various cereals [12,21].

In addition to yield performance, great attention was paid to grain quality. Drought stress causes changes in carbohydrate and nitrogen assimilation rates, a decrease in starch accumulation and an increase in non-reducing sugars which may result in ovary abortion, leading to poor grain set [22,23]. In most of the studies, lower water availability was associated with higher grain protein concentration in durum wheat grains; however, the effects on gluten-forming proteins are somewhat contrasting (e.g., high molecular weight protein subunits and the ratio of glutenin macromolecules) [24]. Nonetheless, severe drought decreases the protein ratio, gluten rate, and sedimentation volume [25,26], hectoliter weight. The decrease in grain carbon (starch) accumulation under water deficit leads to an increase in mineral (ash) concentration (e.g., Ca, Zn, Fe, and Mg) [27,28,29].

Most research on Se application has focused on improving agronomic performances, mitigating abiotic and biotic stresses, and improving nutrient uptake. Nevertheless, enhancing grain quality by Se is generally inferred as an indirect effect of nutrient uptake. Furthermore, most studies have focused on grain biofortification by Se to increase nutrient values for healthy human nutrition [30,31]. To the best of our knowledge, the literature lacks information on the effects of Se on quality proprieties of durum wheat including storage durability and technological values, such as moisture, falling number, protein and gluten contents, and deformation energy value.

Several modes of application of Se might be used including seed coating/soaking, soil application, and foliar spray [13,32]. Se as basal or foliar fertilization is simple and considered as an effective and practical method for plant nutrient supply. Interestingly, foliar fertilization is up to eight times more efficient than soil Se application [33]. This greater efficiency of foliar-applied fertilizers might be ascribed to (1) rapid uptake and assimilation due to application at a later growth stage, (2) less influence of root-to-shoot ratio on translocation to the edible parts of crops, and (3) the avoidance of losses through fixation in soils [34]. Indeed, on average only 12% of soil-applied Se fertilizers are taken up by plants; most Se applied is retained and immobilized in the soil, with very little residual value for subsequent crops [35,36]. This means that repeated applications of Se fertilizers are required for each growth period unless the efficacy of Se fertilizers can be improved. In foliar application, Se ions easily diffuse to epidermal cells as they are transported by the xylem and phloem, hence becoming part of the plant body [37]. Since foliar and root absorption depends on genotype and growing conditions, specific studies are thereby required to assess the effect of these factors on Se efficiency. Therefore, the inclusion of selection for better response to Se in several environments, as a durum wheat breeding objective, in addition to investigations for other agronomic and quality traits, is of great importance. In this context, the aim of this research was to assess (i) the effects of Se supply on two types of germplasm (16 durum wheat advanced lines and nine modern varieties) based on grain yield and grain quality characteristics in two contrasting environments (Kef–Boulifa and Beja) (ii) the genetic variation for Se response that exists in durum wheat breeding programs for potential exploitation.

## 2. Results

### 2.1. Effects of Environments, Se Application, and Cultivars on Grain Yield

The analysis of variance (ANOVA) was performed to investigate the effect of Se treatments on grain yield as well as genetic responses for the two studied environments (Kef–Boulifa and Beja), separately or in combination. Global statistic data showed significant differences (*p* ≤ 0.01) among environments (E), Se treatments (SeTR), and cultivars (C) for this attribute (Table 1). Double and triple interactions (E × SeTR, E × C, SeTR × C, and E × SeTR × C) were also significant (*p* ≤ 0.01). Therefore, this result suggests that the genotypic responses depend on the Se treatment and environment.

Considering each environment (Kef–Boulifa or Beja), the same result was obtained for Se treatment and cultivar effects (*p* ≤ 0.01) (Table 1). Nevertheless, the interaction between the two factors (SeTR × C) was not significant (*p* > 0.05) indicating that the response of each cultivar is stable facing Se treatment.

Globally, the average grain yield was 0.23 kg m^−2^ for all environments under both Se treatments (Table 2). The results showed also that the tested cultivars (i.e., advanced lines and modern varieties) outperformed in the sub-humid region (Beja, 0.32 kg m^−2^) compared to the semi-arid region (Kef–Boulifa, 0.13 kg m^−2^). The achieved mean yields ranged from 0.10 to 0.17 kg m^−2^ at Kef–Boulifa and from 0.25 to 0.42 kg m^−2^ at Beja, using both Se treatments.

**Figure 1:**
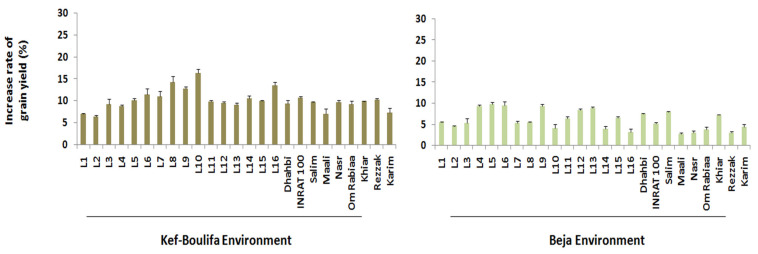
Increase rate of grain yield of 25 durum wheat cultivars after Se foliar supply across the two environments.

Compared to the control treatment, Se foliar supply increased the grain yield by 9.91 and 5.86% under Kef–Boulifa and Beja conditions, respectively (Figure 1). Regarding the two germplasm types, the advanced lines outyielded the modern varieties using both Se treatments in the Kef–Boulifa (0.15 and 0.14 kg m^−2^, respectively) and Beja environment (0.34 and 0.32 kg m^−2^, respectively). Moreover, under both agro-climatic environments, the advanced lines (10.91% and 6.56% increased rate of grain yield, respectively) valorized better the Se foliar application compared to the modern varieties (8.91% and 5.17%, respectively).

Under Kef–Boulifa conditions, the advanced lines, L12, L11, and L5 recorded the highest grain yield and showed a similar trend with the application and non-application of Se (Table 2) which explains the non-significant interaction between Se treatment and cultivars (SeTR × C) (Table 1). A similar finding was obtained for the modern varieties and INRAT 100 followed by Dhahbi and Rezzak exhibited the best yield performances. Nonetheless, these cultivars (i.e., advanced lines and modern varieties) were not necessarily those that better valorized the Se treatment (Figure 1). In fact, L10 (16.36%), L8 (14.29%), and L16 (13.51%) exhibited the highest grain yield increase rates for advanced lines, and INRAT 100 (10.71%), Rezzak (10.28%), and Khiar (9.88%) for modern varieties.

These results were not fully maintained under Beja conditions (Table 2). In fact, L2, L1, and L3 were the top yielding advanced lines under both Se treatments, while Dhahbi, INRAT 100, and Salim were the best performing modern varieties. Otherwise, L5 (9.80%), L6 (9.46%) and L4 (9.35%) showed the highest grain yield increase rates for advanced lines, and Salim (7.97%), Dhahbi (7.48%) and Khiar (7.25%) for modern varieties (Figure 1).

### 2.2. Effects of Environments, Se Application, and Cultivars on Grain Quality Attributes

The ANOVA results revealed significant effects (*p* ≤ 0.05) of environments (E), Se supply (SeTR), and cultivars (C) on all technological traits (i.e., moisture, protein and gluten contents, Zeleny sedimentation index, and deformation energy), except the insignificant effect of SeTR on moisture content and deformation energy. The interactions E × SeTR, E × C, SeTR × C, and E × SeTR × C were also significant (*p* ≤ 0.05) for almost studied attributes (Table 3). For each environment (Kef–Boulifa or Beja), significant variations (*p* ≤ 0.05) were obtained among the different factors (SeTR and C), except the non-significant difference (*p* > 0.05) between cultivars and SeTR × C for gluten content under Kef–Boulifa conditions, and cultivars for moisture content under Beja conditions.

Considering the results of both environments, the average values of moisture, protein and gluten contents, Zeleny sedimentation index, and deformation energy were, respectively, 10.11%, 13.36%, 32.83%, 33.12 cm^3^, and 212.22 10^−4^ J under both Se treatments (Table 4). Notably, the highest values of the three last attributes were observed under Beja conditions (14.13%, 33.51%, 34.22 cm^3^, and 215.34 10^−4^ J, respectively) compared to Kef conditions (12.59%, 32.16%, 32.02 cm^3^, and 209.10 10^−4^ J, respectively). However, the lowest values of moisture content were recorded in the Kef–Boulifa region (9.46%) compared to the Beja region (10.77%).

**Figure 2:**
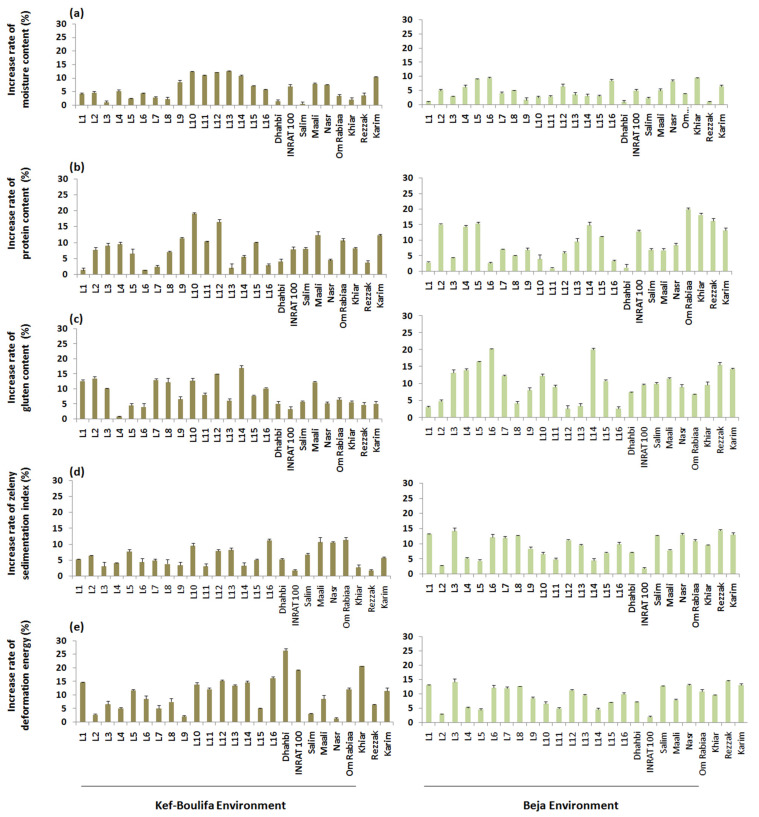
Increase rate of moisture (**a**), protein (**b**) and gluten (**c**) contents, Zeleny sedimentation index (**d**), and deformation energy (**e**) of 25 durum wheat cultivars after Se foliar supply across the two environments.

As expected, Se foliar application induces changes in the durum wheat grain technological characteristics by increasing moisture, protein, and gluten contents, Zeleny sedimentation index, and deformation energy by 5.61%, 8.37%, 8.98%, 5.36%, and 10.10% (Figure 2). In particular, the beneficial Se effect was more marked under Beja conditions for moisture, protein and gluten contents. Otherwise, deformation energy was the best enhanced trait.

Overall, the advanced lines grown in Kef–Boulifa and Beja sites showed better performance for all quality traits compared to the modern varieties either in the presence or absence of Se treatment (Table 4). However, in most cases, the modern varieties valorized better the Se foliar application (e.g., 9.05% increased rate of protein content for both environments) compared to the advanced lines (7.68% increased rate of protein content for both environments) (Figure 2).

For the four grain quality attributes, the tested cultivars responded differently to Se treatments and environmental conditions accounting for significant interactions, such as Se TR × C, E × C, and E × Se TR × C for most traits. Under semi-arid conditions (Kef–Boulifa region), the best advanced lines under stimulator treatment were L3, L5 and L1 for moisture content, L10, L3 and L11 for protein content, L1, L3 and L6 for gluten content, L1, L10 and L11 for Zeleny sedimentation index, and L7, L3 and L16 for deformation energy (Table 4). For modern varieties, the best values were obtained for Maali, Salim and Khiar for moisture content, Dhahbi, Om Rabiaa and Maali for protein content, Maali, Dhahbi and Karim for gluten content, Maali, Dhahbi and Nasr for Zeleny sedimentation index, and Maali, Dhahbi and INRAT 100 for deformation energy.

Under sub-humid conditions (Beja region), the best values recorded for advanced lines under Se treatment were those of L13, L7, L15 and L3 for moisture content, L3, L9 and L15 for protein content, L14, L6 and L15 for gluten content, L2, L1 and L5 for Zeleny sedimentation index, and L2, L14 and L3 for deformation energy (Table 4). Otherwise, Rezzak, Maali and Om Rabiaa for moisture content, Salim, INRAT 100 and Khiar for protein content, Nasri, Dhahbi and Maali for gluten content, Maali, Nasr and Om Rabiaa for Zeleny sedimentation index, and Salim, Maali, and Nasr for deformation energy proved to be the best modern varieties. As shown for grain yield, these lines and modern varieties were not necessarily the cultivars that best valorized the Se application under both conditions (Table 4, Figure 2).

### 2.3. Analysis of ‘Cultivar-Treatment’ Combinations Based on Grain Yield and Quality Attributes

**Figure 3:**
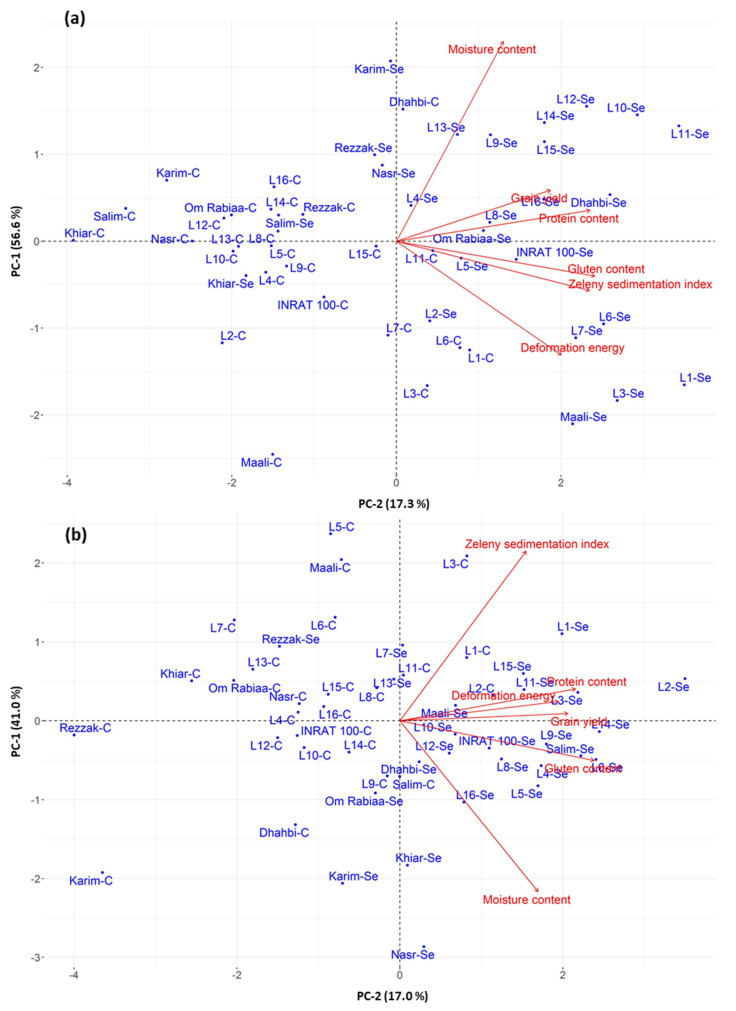
Two-dimensional principal component analysis (PCA) of all combinations (cultivar-treatment) for grain yield and quality attributes under the Kef–Boulifa (**a**) and Beja (**b**) conditions.

The PCA was applied in order to evaluate the relation between cultivars and applied treatments (with or without Se). For the Kef–Boulifa environment, the first and the second principal components (PC-1 and PC-2) accounted for 57% and 17%, respectively, of the total data variance; i.e., their mutual projections (Figure 3 and Table S1). Two groups might be discerned: the first group combined most of the Se-treated cultivars, while the second group was mainly constituted of most untreated cultivars (control). Therefore, the PCA results confirmed the noteworthy beneficial effect of Se on grain yield and quality traits since the Se-treated cultivars were correlated with the studied traits. Interestingly, L11 and L1 seem to combine high yield with good grain quality. In particular, L11 was one of the best performers showing good protein and gluten contents, good Zeleny sedimentation index, and medium deformation energy. Otherwise, L1 had good yield performance (lower than L11), but better quality than L11 (Table 2 and Table 4, Figure 3). These lines were followed by L10, L3, L6, and L12. For the modern varieties, Dhahbi followed by Maali have combined both good yield and good grain quality. When comparing the two types of germplasm, some advanced lines (L1, L11, L10, and L3) have noted superiority for the studied traits over modern varieties.

For the Beja environment, the first two axes (PC-1 and PC-2) presented 58% of the total variability (Figure 3, Table S1). The distribution of ‘cultivar-treatment’ combinations followed the same trend as that obtained under semi-arid conditions indicating the superior performance of Se-treated cultivars compared to that of untreated cultivars. Under sub-humid conditions, L2, L14, L6, and L3 achieve both good yield and good grain quality. Otherwise, Salim followed by INRAT 100 were the best cultivars for the modern varieties. Notably, L2, L14, and L6 showed great superiority when considering advanced lines and commercial varieties (Table 2 and Table 4, Figure 3).

## 3. Discussion

### 3.1. Se Fertilization and Its Role in Improving Grain Yield

The present investigation highlighted a positive effect of Se application on durum wheat grain yield across the two environments. This is in line with the findings of Curtin et al. [18], Ducsay et al. [38] and De Vita et al. [31]. According to Nawaz et al. [39], Se foliar application promoted growth and yield of wheat and can reach 24%. However, Radawiec et al. [40] depicted that Se fertilization did not affect the grain yield. This beneficial effect might be attributed to several indirect actions of Se. In fact, yield gain due to Se uptake is partially a result of increased chlorophyll concentrations, sustained photosynthetic activity, maintenance of leaf architecture for effective light interception, and regulation of respiration [16,41]. Se plays also a magnificent role in improving water use efficiency in the roots, curtailing water loss from tissues [42] and delaying senescence [43]. In addition, Se acts as an important component of antioxidant enzymes, which could counteract oxidative stress by protecting the cell membrane against lipid peroxidation [44,45,46]. Exo-applied Se can also contribute to the uptake of more nutrients by plants, as reported in various cereals [47,48].

This research indicated that the Se effect was strongly dependent on the environment with better results at the Kef–Boulifa site compared to the Beja site. This meaningful difference might be due to the weather conditions of each experimental site in terms of rainfall and temperature. The rainfall (amount and distribution) and temperature varied considerably between the two environments (Kef–Boulifa and Beja), especially during the critical periods of crop development (e.g., anthesis and grain filling). In fact, the Beja site belongs to the sub-humid zone with an average rainfall during the durum wheat growing season (November to June 2019/20) of 409.2 mm. However, Kef–Boulifa is located in a semi-arid region with an average rainfall of 281.6 mm during the same cropping season (2019/20). Compared to the Beja site, the Kef–Boulifa site stood out by having a cold winter (10.9, 7.7, and 10.3 °C in December, January, and February, respectively) and a cold spring (13.4 °C in March) which are expected to increase the length of the development cycle; however, some cold damages may have occurred which can explain the superiority of grain yield in the Beja compared to the Kef region. The difference obtained for the Se effect on grain yield between the two environments might be also attributed to the physicochemical soil characteristics (e.g., soil texture, pH, etc.). In fact, Lyons et al. [49] noted that soil texture and its physicochemical properties influence the relative effectiveness of Se supply in improving the grain yield crops. For instance, it is well known that Se foliar application enhances the water use efficiency that might be influenced by soil characteristics. The same trend might be observed for nutrient uptake. Although the Beja site has more favorable conditions (e.g., higher rainfall and higher amounts of P, K, and organic matter), the effect of Se fertilization on grain yield seems to be more pronounced in marginal environments, frequently exposed to abiotic constraints (e.g., water-deficit and salinity). Thereby, Se might be considered as a reliable nutrient to induce plant tolerance, in turn, making this element more prospective in helping durum wheat plants to acclimate successfully to semi-arid conditions.

### 3.2. Se Fertilization and Its Role in Improving Grain Quality Attributes

In this study, Se application enhanced the grain quality. The protein content is among the most useful indicator for characterizing durum wheat cultivars. Moreover, protein content composition has an important impact on the durum wheat processing quality. In our case, Se increased the protein content from 12.09 to 13.10% under semi-arid conditions and from 13.46 to 14.81% under sub-humid conditions. This is in line with the results of Radawiec et al. [40] on spring wheat. The obtained values are indicators of good grain quality since the protein content that made satisfactory pasta or leavened bread should be at least ≥13% [50]. An equally important quality characteristic is gluten content, which has a direct effect on the rheology (viscoelasticity) and the properties of cooked pasta [51,52,53]. Se application led to a gluten content of 33.50% and 35.30% at the Kef–Boulifa and Beja sites, respectively. Similar to protein content, these values are acceptable in terms of good technological quality since they are ≥17% [50]. Otherwise, the Zeleny sedimentation index, which to a large extent determines the baking value of wheat flour, should not be lower than 20 cm^3^ [54] and preferably higher than 30 cm^3^ [55]. A sedimentation index value above 50 cm^3^ is typical of high-protein wheat containing the so-called “strong gluten” [56]. The obtained results (33.02 and 35.05 cm^3^ under Kef–Boulifa and Beja conditions, respectively) meet the requirements of grain intended for baking purposes for this feature. According to Radawiec et al. [40], Se supply did not affect the gluten content and Zeleny sedimentation index. Another relevant component of dough rheological properties is the deformation energy, predicting flour processing behavior and sufficient firmness of well-cooked semolina products [57]. The durum semolina-flour should have a deformation energy value ≥200 10^−4^ J [50]. In this study, the values were recorded for this quality trait (221.42 and 225.83 10^−4^ J under Kef–Boulifa and Beja conditions, respectively) with Se addition being an indicator of good technological quality.

The positive outcomes of Se supply on quality attributes might be related to the beneficial effect of Se on chlorophyll content and net photosynthetic rate during the grain-filling stage, which could positively influence the grain quality [58]. This result might be also explained by the key role of this element to maintain the balance of N and C. This is important in ensuring high grain quality, especially under stressed conditions. In fact, the balance of C and N plays a crucial role in the synthesis of gliadin and glutenin, which are the main components of gluten in wheat [59]. Previous reports showed that Se uptake has cascading effects on C, N, and S uptake. In fact, several authors reported a positive effect of Se on N uptake and N utilization efficiency in wheat plants [60,61], probably due to the increase of the nitrate reductase activity [61]. Thereafter, increased N accumulation in wheat grains will enhance protein content [62,63], resulting in an increase in both gliadins and glutenins [64,65] that indirectly affects the rheological properties and grain quality of wheat [12,21].

The grain quality is greatly influenced by management and the environment [66,67]. Taking into account that the protein and gluten contents are the most relevant parameters for the quality of durum wheat [68,69], the beneficial Se effect was more pronounced under Beja conditions, unlike grain yield. It is well documented that protein content is a typical quantitative trait controlled by complex genetic arrangements under the high influence of pedo-climatic factors [70,71]. Water management and nitrogen application are important factors that determine wheat grain protein quality. Water deficit significantly increases the protein content mainly due to higher rates of accumulation of grain N and lower rates of accumulation of carbohydrates; on the other hand, irrigation may decrease protein content by dilution of N with carbohydrates [72]. Later, Carucci et al. [60] and Lupini et al. [73] stated, however, that water stress decreased N use efficiency, N uptake efficiency, and N utilization efficiency, except for some durum wheat genotypes. Therefore, the effect of water and N assimilation interaction on protein content seems to imply a complex framework of multiple physiological processes that might be controlled by genetic factors. Otherwise, protein content is strongly associated with high temperatures during the grain-filling stage [74]. Similarly, weaker gluten quality was reported in the seasons having cooler and wetter weather [75]. Thereby, the higher mean temperature recorded at the Beja site might be one of the reasons for the higher increase in protein and gluten contents compared to those of the Kef–Boulifa site. As mentioned in several reports [76,77], strong correlations between protein content, gluten content, Zeleny sedimentation index, and deformation energy were obtained in this study for each environment (data not shown). Therefore, the environmental conditions of each site could have approximately the same influence on these grain quality attributes.

### 3.3. Cultivar Responses to Se Fertilization

A genotypic variation was noted for the tested germplasm (advanced lines and modern varieties) either in the presence or absence of Se treatment within and among the two studied zones. Overall, the Se-treated advanced lines displayed the highest grain yield under Kef–Boulifa and Beja conditions. Interestingly, L12, L11, L5, INRAT 100 and Dhahbi outyielded the rest of the cultivars in the semi-arid region, while L2, L1, L3, Dhahbi and INRAT 100 were the best yielders in the sub-humid region.

Although advanced lines showed better grain quality than modern varieties under both Se treatments, this last type of germplasm performed better with the Se foliar application. Similar to grain yield, the cultivars that showed good quality under semi-arid conditions were not the same under sub-humid conditions. All obtained results suggest that there is a specific adaptation of cultivars to each environment in addition to a specific interaction between Se and cultivars. In fact, it is also worth noting that Se treatment boosted the yield or the grain quality of some cultivars that are not necessarily the best performers.

Genotype-specific Se responses were also noted by Lyons et al. [78] among modern elite wheat in controlled field trials. This genotypic variability for yield and quality attributes might be explained by the differential ability to sequester Se in non-toxic organic compounds. Part of this variation may be due also to differences in transport processes and physiological responses of genotype to this element [79]. Previous studies have shown that the improvement of stress tolerance is related to a better accumulation of Se [30]. Iqbal et al. [80] suggested that involving genetic improvements to enhance Se accumulation potential would be a sustainable approach in long term.

In breeding programs, breeders aim to select cultivars that combine high yield with good grain quality. Therefore, under a semi-arid climate, L11, L1, Dhahbi, and Maali were the best advanced lines and modern varieties, respectively. Otherwise, under sub-humid climate, L2, L14, L6, L3, Salim, and INRAT 100 combined both good yield and good technological quality. Comparing the two types of germplasm, some advanced lines (e.g., L1, L11, L10 and L3 for the Kef–Boulifa environment, and L2, L14 and L6 for the Beja environment) have noted superiority for the studied traits compared to the modern varieties. These promising selections are recommended to be developed in the specific ecological zone in Tunisia for sustainable production.

## 4. Materials and Methods

### 4.1. Genetic Material and Sites Description

Sixteen genetically diverse durum wheat (*Triticum turgidum* L. ssp. *durum* [Desf.] Husn.) promising lines, sourced from the national breeding program and selected for grain yield potential and foliar diseases across several sites, were used in this study (Table 5). In addition, nine Tunisian modern varieties (Dhahbi, INRAT 100, Salim, Maali, Nasr, Om Rabiaa, Khiar, Rezzak, and Karim), registered in the national catalog of durum wheat varieties and marketed in all Tunisian regions, were also chosen. The vegetal material was evaluated under field conditions of two different climate locations representative of major rainfed cultivated durum wheat growing areas in Tunisia, namely Kef–Boulifa and Beja. The experimental area of Kef–Boulifa (36°11′10″ N; 8°42′00″ E, at 532 m) has a Mediterranean semi-arid climate (Table 6), and the soil of the experimental station is classified as clay-loam [81] with 98.00 ppm available N (Kjeldahl method), 16.53 ppm available P (Olsen method), 510.00 ppm available K (ammonuim acetate method), 1.41% of organic matter (Walkley-Black method), and pH = 7.91. Otherwise, Beja (36°43′ N; 9°12′ E, at 161 m) is a typically sub-humid region and the soil of the experimental station is identified as vertic [81] with 87.53 ppm available N, 80.00 ppm available P, 621 ppm available K, 2.10% of organic matter, and pH = 7.00. The two experimental sites (Kef–Boulifa and Beja) represent two different environments, which are the combination of one cropping season (2019/20) and two locations.

### 4.2. Se Treatments and Field Management

The experiments were performed in Kef–Boulifa and Beja sites during the growing season 2019/20. The experimental design was arranged in randomized complete block (RCB) for each site with three replicates per treatment (*n* = 3). Two Se treatments were adopted in this study: with Se and without Se (control). For each site, six blocks (in total 1080 m^2^) for the two treatments (i.e., with and without Se) were subdivided each into 25 plots of 7.2 m^2^ (in total 75) containing six rows of 6 m length, with 0.2 m inter-row spacing and 0.5 m inter-plot spacing. Seeds were sown with a density of 350 grains m^−2^.

Sodium selenite (Na_2_SeO_3_), an inorganic compound was used as a Se source and applied as an aqueous Na_2_SeO_4_ solution, which was uniformly mixed with water. Se treatment was implemented by foliar application, under sunny and dry conditions, using 5.00 g Na_2_SeO_4_ ha^−1^ (spraying volume per hectare was 300 L) at two vegetative growth stages, at the beginning of tillering (Z21, 40 and 50 days after sowing in the Beja and Kef–Boulifa sites, respectively) and at the end of tillering (Z29, 70 and 85 days after sowing in Beja and Kef–Boulifa sites, respectively) [82]. Therefore, the total fertilizer dose was 10.00 g Se ha^−1^ [34]. Basal fertilization of 100 kg ha^−1^ of di-ammonium Phosphate was provided at sowing, followed by three split applications of ammonium nitrate (33.5% N) of 100 kg ha^−1^ each at early tillering (Z13), at stem elongation (Z16), and at 2nd node visible (Z32) [82]. Weeds were controlled by a mix of mechanical interventions and chemical control using an herbicide at the 2–3 leaf stage (Z12–13) [82].

### 4.3. Measured Agronomic and Grain Quality Traits

At maturity, grain yield (kg m^−2^) was recorded for each advanced line and modern variety in Kef–Boulifa and Beja environments. In addition, the spectroscopic method (NIRS) that uses the near-infrared region of the electromagnetic spectrum (from ~800 nm to 2500 nm), was employed to determine four technological quality traits. The NIRS provides scorings for moisture content (%), protein content (%), gluten content (%), Zeleny sedimentation index (cm^3^), and deformation energy (10^−4^ J).

### 4.4. Statistical Data Analysis

When data followed a normal distribution, the ANOVA for an RCB design was used to quantify the effect of all factors used for the statistical analysis, i.e., environments, Se treatments, and cultivars on the measured grain yield and quality attributes. Comparisons of datasets were subjected to Tukey’s multiple range test at a 5% significance level. Statistical analysis was performed using SPSS software ver. 16.0 (IBM SPSS Statistics. SPSS for Windows, Version 16.0. Chicago, IL, USA, SPSS Inc., 2007). Otherwise, to describe the relationship between ‘cultivar-treatment’ combinations, principal component analysis (PCA) was performed using R statistical software version 4.0 (The R Foundation for Statistical Computing).

## 5. Conclusions

Se foliar application can be considered as a safe way of increasing grain yield, especially in the Kef–Boulifa (semi-arid zone) environment. In terms of grain quality, Se fertilization showed, however, better results under the Beja (sub-humid zone) environment. Genotype-specific Se responses were obtained within and among the two studied zones. Overall, the advanced lines showed a better Se efficiency and valorization on grain yield under both environments. The same result was partly maintained for the quality; i.e., modern varieties valorized Se foliar supply better. Based on these study outcomes, the outperformer modern varieties (e.g., Dhahbi and Maali for the semi-arid region, and Salim and INRAT 100 for the sub-humid region) might be recommended to farmers that should adopt Se fertilization. Moreover, the outperformer advanced lines (e.g., L11 and L1 for semi-arid region, and L2, L14, L6 and L3 for sub-humid region) might be considered as promising lines. The obtained genetic variation for Se supply response might be exploited by breeders to develop varieties that will efficiently use the applied Se for improved yield and grain quality, especially in marginal conditions.

## Figures and Tables

**Table 1:** Analysis of variance (*F* values) for grain yield of 25 durum wheat cultivars across the two environments.

Sources of Variance	df	*F*
E	1	19,454.70 **
SeTR	1	292.45 **
C	24	28.92 **
E × SeTR	1	55.34 **
E × C	24	22.91 **
SeTR × C	24	3.15 **
E × SeTR × C	24	3.13 **
Kef–Boulifa Environment		
SeTR	1	42.31 **
C	24	6.60 **
SeTR × C	24	0.84 ^ns^
Beja Environment		
SeTR	1	335.74 **
C	24	49.65 **
SeTR × C	24	5.96 ^ns^

df, degree freedom; E; environments; SeTR, selenium treatments; C, cultivars; ns, non significant; **, significant at 0.01.

**Table 2:** Grain yield of 25 durum wheat cultivars across the two environments.

Cultivars	Grain Yield (kg m^−2^)			
	Kef–Boulifa Environment		Beja Environment	
	Without Se	With Se	Without Se	With Se
Advanced Lines				
L1	0.15 b	0.16 ab	0.36 b	0.38 b
L2	0.12 d	0.13 d	0.40 a	0.42 a
L3	0.12 d	0.13 d	0.34 b	0.36 b
L4	0.13 cd	0.14 cd	0.31 cd	0.34 bc
L5	0.15 b	0.16 ab	0.31 cd	0.34 bc
L6	0.14 bc	0.16 ab	0.30 cd	0.34 bc
L7	0.13 cd	0.15 bc	0.27 d	0.29 e
L8	0.14 bc	0.16 ab	0.33 b	0.35 bc
L9	0.14 bc	0.16 ab	0.31 cd	0.34 bc
L10	0.13 cd	0.15 bc	0.29 d	0.30 de
L11	0.15 b	0.17 a	0.32 bc	0.34 bc
L12	0.16 a	0.17 a	0.30 cd	0.33 cd
L13	0.12 d	0.14 cd	0.30 cd	0.33 cd
L14	0.13 cd	0.14 cd	0.33 b	0.35 bc
L15	0.13 cd	0.14 cd	0.27 d	0.29 e
L16	0.13 cd	0.15 bc	0.29 d	0.30 de
Mean	0.14	0.15	0.32	0.34
Modern Varieties				
Dhahbi	0.13 cd	0.15 bc	0.33 b	0.35 bc
INRAT 100	0.14 bc	0.15 bc	0.33 b	0.34 bc
Salim	0.13 cd	0.14 cd	0.32 bc	0.35 bc
Maali	0.13 cd	0.14 cd	0.30 cd	0.30 de
Nasr	0.13 cd	0.14 cd	0.31 cd	0.32 de
Om Rabiaa	0.12 d	0.13 d	0.32 bc	0.33 cd
Khiar	0.10 e	0.11 e	0.25 e	0.27 f
Rezzak	0.13 cd	0.15 bc	0.30 cd	0.31 de
Karim	0.12 d	0.13 d	0.30 cd	0.31 de
Mean	0.13	0.14	0.30	0.32
Global Mean	0.13	0.14	0.31	0.33

Different letters show the significant differences at 0.05 (Tukey test).

**Table 3:** Analysis of variance (*F* values) for grain quality attributes of 25 durum wheat cultivars across the two environments.

Sources of Variation	df	Moisture Content	Protein Content	Gluten Content	Zeleny Sedimentation Index	Deformation Energy
E	1	1308.23 ***	510.85 ***	41.49 ***	32.49 *	22.09 ***
SeTR	1	0.80 ^ns^	359.70 ***	63.95 ***	42.95 **	0.38 ^ns^
C	24	3.70 ***	10.63 ***	1.78 *	9.16 *	11.66 ***
E × SeTR	1	68.26 ***	16.97 ***	5.61 *	8.14 *	17.75 ***
E × C	24	3.40 ***	7.34 ***	1.26 ^ns^	10.26 **	10.71 ***
SeTR × C	24	4.50 ***	4.17 ***	0.70 ^ns^	5.13 **	4.98 ***
E × SeTR × C	24	3.23 ***	3.89 ***	1.03 ^ns^	0.18 ^ns^	10.38 ***
Kef–Boulifa Environment						
SeTR	1	27.89 ***	264.15 ***	4.89 *	7.23 *	130.06 ***
C	24	8.55 ***	42.70 ***	1.44 ^ns^	12.52 *	156.74 ***
SeTR × C	24	11.28 ***	11.96 ***	0.76 ^ns^	2.43 *	224.54 ***
Beja Environment						
SeTR	1	32.09 ***	695.29 ***	82.82 ***	29.96 **	239.72 ***
C	24	1.54 ^ns^	26.29 ***	2.15 **	4.11 **	330.24 ***
SeTR × C	24	1.84 *	16.42 ***	1.93 **	3.64 **	156.60 ***

df, degree freedom; E; environments; SeTR, selenium treatments; C, cultivars; ns, non significant; *, **, *** significant at 0.05, 0.01, and 0.001.

**Table 4:** Grain quality attributes of 25 durum wheat cultivars across the two environments.

**Cultivars**	**Moisture Content (%)**				**Protein Content (%)**				**Gluten Content (%)**			
	**Kef–Boulifa Environment**		**Beja Environment**		**Kef–Boulifa Environment**		**Beja Environment**		**Kef–Boulifa Environment**		**Beja Environment**	
	**Without Se**	**With Se**	**Without Se**	**With Se**	**Without Se**	**With Se**	**Without Se**	**With Se**	**Without Se**	**With Se**	**Without Se**	**With Se**
Advances Lines												
L1	8.75 c	9.12 cd	10.82 a	10.93 cd	12.69 a	12.86 bc	14.86 a	15.31 a	34.31 a	39.30 a	33.89 ab	34.95 c
L2	8.77 c	9.18 cd	10.77 a	11.33 ab	11.61 ab	12.57 bc	12.23 cd	14.41 bc	30.23 ab	34.97 bc	34.23 a	35.96 bc
L3	8.88 c	8.96 d	10.53 ab	10.84 cd	13.08 a	14.38 a	15.67 a	16.38 a	33.10 a	36.84 b	30.27 c	34.86 c
L4	9.02 bc	9.51 bc	10.43 ab	11.11 ab	12.12 ab	13.39 ab	12.89 bc	15.06 ab	30.75 ab	31.00 bc	32.85 bc	38.21 a
L5	8.88 c	9.10 cd	10.19 b	11.19 ab	12.02 ab	12.85 bc	12.56 bc	14.85 ab	31.08 ab	32.57 bc	30.87 c	36.95 bc
L6	8.87 c	9.27 cd	10.22 b	11.28 ab	13.02 a	13.19 ab	14.67 a	15.08 ab	34.83 a	36.31 b	31.52 c	39.48 a
L7	9.15 bc	9.41 bc	10.18 b	10.60 c	12.40 a	12.69 bc	14.59 a	15.70 a	31.00 ab	35.64 b	31.23 c	35.61 bc
L8	9.16 bc	9.37 bc	10.64 ab	11.20 ab	11.93 ab	12.83 bc	14.08 ab	14.83 ab	30.07 ab	34.28 bc	33.07 ab	34.51 c
L9	9.18 bc	10.03 ab	10.79 a	10.97 ab	11.54 ab	13.01 ab	14.85 a	15.95 a	29.83 ab	31.94 cd	34.60 a	37.63 ab
L10	9.06 bc	10.33 a	10.80 a	11.09 ab	12.01 ab	14.83 a	13.34 ab	13.90 bc	28.75 b	32.97 bc	33.10 ab	37.75 ab
L11	9.18 bc	10.31 a	10.79 a	11.11 ab	12.47 a	13.89 a	14.70 a	14.87 ab	32.84 ab	35.71 b	34.14 a	37.55 ab
L12	8.99 c	10.23 a	10.38 ab	11.09 ab	11.04 bc	13.22 ab	13.08 bc	13.90 bc	29.45 bc	34.66 bc	34.71 a	35.64 bc
L13	9.05 bc	10.34 a	10.10 b	10.46 c	12.47 a	12.72 bc	12.60 bc	13.94 bc	30.95 ab	32.97 bc	34.31 a	35.51 bc
L14	9.32 ab	10.44 a	10.63 a	10.96 ab	12.69 a	13.41 ab	12.94 bc	15.20 a	29.47 bc	35.49 b	31.60 c	39.49 a
L15	9.48 a	10.20 ab	10.27 b	10.60 c	12.41 a	13.78 ab	14.02 ab	15.78 a	32.26 ab	34.98 bc	34.77 a	38.98 a
L16	9.51 a	10.07 ab	10.68 a	11.66 a	12.65 a	13.02 ab	13.85 ab	14.33 bc	29.47 bc	32.83 bc	34.63 a	35.59 bc
Mean	9.08	9.74	10.51	11.03	12.26	13.29	13.81	14.97	31.15	34.53	33.11	36.79
Modern Varieties												
Dhahbi	9.97 a	10.11 ab	10.80 a	10.89 cd	13.49 a	14.06 a	12.79 bc	12.95 c	31.65 ab	33.37 bc	34.09 a	36.85 bc
INRAT 100	8.88 c	9.53 bc	10.56 ab	11.11 ab	12.11 ab	13.13 ab	13.87 ab	15.91 a	31.32 ab	32.39 bc	31.46 c	34.83 c
Salim	9.15 bc	9.19 cd	10.83 a	11.09 ab	10.74 bc	11.66 c	14.97 a	16.08 a	29.12 bc	30.93 bc	31.68 c	35.19 bc
Maali	8.21 c	8.90 d	10.06 b	10.59 c	11.71 ab	13.35 ab	14.05 ab	15.08 ab	30.09 ab	34.32 bc	31.33 c	35.43 bc
Nasr	9.18 bc	9.92 ab	10.56 ab	11.51 a	10.82 bc	11.32 c	11.98 cd	13.08 c	30.98 ab	32.70 bc	34.36 a	37.77 ab
Om Rabiaa	9.36 ab	9.68 bc	10.47 ab	10.88 cd	12.09 ab	13.52 ab	12.41 cd	15.52 a	30.90 ab	33.05 bc	28.93 cd	31.05 d
Khiar	9.09 bc	9.27 cd	10.59 ab	11.68 a	10.95 bc	11.92 bc	12.89 bc	15.76 a	28.48 b	30.19 bc	30.55 c	33.83 cd
Rezzak	9.30 ab	9.64 bc	10.44 ab	10.53 c	11.92 ab	12.37 bc	12.03 cd	14.37 bc	30.61 ab	32.15 bc	23.77 d	28.15 d
Karim	9.28 ab	10.35 a	10.82 a	11.57 a	11.39 ab	12.97 bc	11.27 d	12.97 c	31.40 ab	33.09 bc	26.76 cd	31.20 d
Mean	9.19	9.84	10.51	11.04	11.92	12.92	13.10	14.66	30.51	32.47	30.33	33.81
Global Mean	9.13	9.79	10.51	11.03	12.09	13.10	13.46	14.81	30.83	33.50	31.72	35.30
**Cultivars**	**Zeleny Sedimentation Index (cm^3^)**								**Deformation Energy (10^−4^ J)**			
	**Kef–Boulifa Environment**				**Beja Environment**				**Kef–Boulifa Environment**		**Beja Environment**	
	**Without Se**		**With Se**		**Without Se**		**With Se**		**Without Se**	**With Se**	**Without Se**	**With Se**
Advances Lines												
L1	34.31 a		36.15 a		35.95 a		36.89 a		209.49 b	245.06 b	185.85 d	213.86 d
L2	30.11 cd		32.17 cd		34.87 a		37.12 a		219.48 b	225.80 c	277.83 a	285.75 b
L3	33.10 a		34.11 b		34.86 a		36.43 a		238.34 a	255.19 a	240.96 bc	280.74 b
L4	30.71 cd		32.00 cd		33.21 ab		34.34 bc		200.23 c	210.96 c	224.85 c	237.01 c
L5	30.08 cd		32.57 cd		33.95 bc		36.89 a		188.25 c	213.27 c	253.14 b	264.83 bc
L6	32.83 ab		34.31 b		34.48 a		35.23 ab		221.65 b	242.19 b	224.61 c	255.83 bc
L7	32.00 ab		33.64 bc		34.61 a		35.65 ab		248.68 a	261.86 a	150.99 e	171.37 e
L8	31.07 bc		32.28 cd		33.51 bc		34.54 bc		194.52 c	210.24 c	197.66 d	226.28 c
L9	30.83 cd		31.90 cd		32.63 cd		34.14 bc		218.88 b	223.48 c	214.00 c	233.64 c
L10	31.65 bc		34.97 b		33.75 bc		35.54 ab		188.99 c	219.12 c	196.43 d	210.10 d
L11	33.64 a		34.71 b		35.55 a		36.64 a		196.16 c	223.34 c	172.04 de	180.83 de
L12	30.11 cd		32.66 cd		32.64 cd		34.71 bc		185.51 c	218.83 c	189.32 d	213.11 d
L13	29.35 d		31.97 d		33.51 bc		34.31 bc		194.03 c	224.10 c	190.35 d	210.28 d
L14	30.47 cd		31.49 cd		32.49 cd		34.60 bc		196.88 c	230.58 bc	272.50 a	285.36 b
L15	32.26 ab		33.98 b		32.98 cd		34.77 bc		202.32 bc	213.17 c	257.67 b	277.07 b
L16	29.16 d		32.83 cd		34.59 a		35.63 ab		210.95 b	251.42 a	167.23 de	185.47 de
Mean	31.36		33.23		33.97		35.46		207.15	229.29	213.46	233.22
Modern Varieties												
Dhahbi	32.47 ab		34.23 b		31.85 d		34.09 bc		180.43 c	244.76 b	179.00 de	192.54 d
INRAT 100	32.42 ab		32.97 cd		32.83 cd		34.46 bc		197.95	244.58 b	184.05 d	187.69 de
Salim	29.23 d		31.34 cd		32.19 cd		33.68 c		176.72 cd	182.30 d	266.00 a	304.32 a
Maali	31.39 bc		35.12 a		33.43 bc		35.56 ab		240.35 a	263.03 a	263.11 a	285.59 b
Nasr	30.12 cd		33.65 bc		34.43 a		35.36 ab		187.49 c	189.99 d	199.01 d	228.95 c
Om Rabiaa	29.32 d		33.05 bc		31.05 d		34.93 bc		196.74 c	223.74 c	190.17 d	213.40 d
Khiar	30.34 cd		31.19 cd		33.83 bc		34.55 bc		165.76 d	208.74 c	159.77 e	176.67 e
Rezzak	32.61 ab		33.15 bc		32.15 cd		34.77 bc		171.21 d	182.83 d	147.89 e	172.90 e
Karim	29.40 d		31.15 cd		30.20 d		32.76 c		161.06 d	181.97 d	177.34 de	203.92 d
Mean	30.71		32.81		32.82		34.64		186.41	213.55	196.26	218.44
Global Mean	31.03		33.02		33.40		35.05		196.78	221.42	204.86	225.83

Different letters show the significant differences at 0.05 (Tukey test).

**Table 5:** List and pedigree of durum wheat advanced lines and modern varieties used in the study.

Genetic Material		Pedigree
N°	Name	
Advanced Lines		
1	L1	Karim/4/BD2337//D68-8-6A-3A/Karim“S”/3/Src2/Src1
2	L2	Site/3×Musk-4//Nasr/3/Maali
3	L3	Dipper/3/Hui//Cit71/Cii/4/Chen/Altar84/6/Srn2/Bisu/4/Khp/D31708//Khp/3/Corm/5/Site/3×Musk-4/7/Lgt3/4/Bcr/3/Ch1//Gta/Stk
4	L4	Somat-4/Silver-1//Site/3×Musk-4/3/Salim
5	L5	Site/3×Musk-4//Salim/4/Bcr/Guerou-1/3/Minimus-6/Plata-16//Immer/5/Maali
6	L6	Salim//D68-8-6a-3a/D68-8-93a-1a
7	L7	Maali/Zeina-4/5/Somo“s”/Stn“s”/3/Tez“s”/Yav79//Hui“s”/4/Chen/Altar84
8	L8	Maali/Zeina-4/5/Somo“s”/Stn“s”/3/Tez“s”/Yav79//Hui“s”/4/Chen/Altar84
9	L9	Site/3×Musk-4//Salim/4/Bcr/Guerou-1/3/Minimus-6/Plata-16//Immer/5/Maali
10	L10	Karim//Cado/Boomer-33
11	L11	Karim/Grecale//Salim
12	L12	Salim/Neodur
13	L13	Maali/4/Stot//Altar 84/Ald/3/Patka_7/Yazi_1/5/Altar 84//Fd8419-126-1-2/ Razzak/3/Krf-Dw/Baladia Hamra=cdss07y00659t-W-1b-12b-7b-0b
14	L14	Maali/4/Arment//Srn_3/Nigris_4/3/Canelo_9.1/5/Altar84//Fd8419-126-1-2/Razzak/3/Krf-Dw/BaladiaHamra=Cdss07y00661t-L-2b-3b-14b-0b
15	L15	Grecale/7/Ainzen-1/6/2×Cmh82a.1062/3/Gdovz394//Sba81/Plc/4/Aaz-1/Crex/5/Hui//Cit71/Cii/8/Maali
16	L16	Grecale/7/Ainzen-1/6/2×Cmh82a.1062/3/Gdovz394//Sba81/Plc/4/Aaz-1/Crex/5/Hui//Cit71/Cii/8/Maali
Modern Varieties		
17	Dhahbi	Karim/4/BD2337//D68-8-6A3A/Karim“S”/3/Src2/Src1
18	INRAT 100	Maali/8/Green_2/Himan_12//Ship_1/7/Eco/Cmh76a.722//Bit/3/Altar84/4/Ajaia_/5/Kjove_1/6/Malmuk_1/Serrator_1/9/Salim/5/Sula/Aaz_5//Chen/Altar84/3/Ajaia_12/F3local(Sel.Ethio.135.85)//Plata_13/4/Arment//Srn_3/Nigris_4/3/Canelo_91
19	Salim	ALTAR84//FD8419-126-1-2/Razzak/3/Krf/Baladia Hamra
20	Maali	CMH80A.1060/4/TTURA/CMH74A.370//CMH77.774/3/YAV79/5/Razzak/6/DACK“S”/YEL“S”//Khiar
21	Nasr	GdoVZ512/Cit//Ruff/Fg/3/Pin/Gre//TrobICD85-1340-ABL-6AP-0TR-10b-3b-0b
22	Om Rabiaa	JoC69/Haurani
23	Khiar	CHEN“S”/ALTAR84
24	Rezzak	DMX69-331/Karim
25	Karim	21563/AA“S”//FG“S”

**Table 6:** Climatic variations in Kef–Boulifa and Beja sites during 2019/20 cropping season.

	Environments	Total	Oct	Nov	Dec	Jan	Feb	Mar	Apr	May	Jun
Rainfall (mm)	Kef–Boulifa	281.6	14.2	39.8	50.6	9.2	3	66.2	70.9	1.4	26.3
	Beja	409.2	30.4	68.6	74.2	20.2	2.6	101.7	105.5	1.6	4.4
	Mean	345.4	108.4	60.7	52.9	127.4	61.9	95.9	40.3	103.2	1.6
Temperature (°C)	Kef–Boulifa	131.8	17.8	13.6	10.9	7.7	10.3	13.4	14.8	17.4	25.6
	Beja	150.9	18.5	17.1	15.6	13.4	12.9	16.6	13.2	17.3	26.3
	Mean	141.3	18.1	15.3	13.2	10.5	11.6	15.0	14.0	17.3	25.9
Evapotranspiration (mm month^−1^)	Kef–Boulifa	150.3	145.7	105.0	80.6	80.6	106.4	148.8	186.0	235.6	264.0
	Beja	145.9	148.8	93.0	74.4	65.1	106.4	139.5	183.0	254.2	249.0
	Mean	148.1	147.3	99.0	77.5	72.9	106.4	144.2	184.5	244.9	256.5

## Data Availability

Available upon reasonable request.

## Supplementary Materials

The following are available online at https://www.mdpi.com/article/10.3390/plants11111437/s1: Table S1—Correlation of the grain yield and quality traits with the first two discriminant axes of PCA.
