# Supplementary material for: Screening of Durum Wheat Cultivars for Selenium Response under Contrasting Environments, Based on Grain Yield and Quality Attributes
Source: Plants (Basel). 2022 May 28;11(11):1437. doi: 10.3390/plants11111437 (PMC9183021; doi:10.3390/plants11111437)
Supplement: Supplementary file 1 [file plants-11-01437-s001.zip › plants-1570405-supplementary.pdf]

Supplementary Material

Table S1. Correlation of the grain yield and quality attributes with the first two discriminant axes of PCA.

Traits	PC-1	PC-2
**Kef-Boulifa Environment**		
Grain Yield	0.366	0.208
Moisture Content	0.254	0.815
Protein Content	0.461	0.126
Gluten Content	0.472	-0.142
Zeleny Sedimentation Index	0.460	-0.201
Deformation Energy	0.392	-0.464
**Beja Environment**		
Grain Yield	0.424	0.030
Moisture Content	0.349	-0.692
Protein Content	0.443	0.129
Gluten Content	0.490	-0.160
Zeleny Sedimentation Index	0.319	0.687
Deformation Energy	0.400	0.078
